# An interactive web application for exploring systemic lupus erythematosus blood transcriptomic diversity

**DOI:** 10.1093/database/baae045

**Published:** 2024-05-28

**Authors:** Eléonore Bettacchioli, Laurent Chiche, Damien Chaussabel, Divi Cornec, Noémie Jourde-Chiche, Darawan Rinchai

**Affiliations:** B Lymphocytes, Autoimmunity and Immunotherapies, UMR 1227, Univ Brest, Inserm, Brest 29200, France; Brest University Hospital, Brest 29200, France; Department of Internal Medicine, Hôpital Européen, Marseille 13003, France; Translational Medicine Division, Research Branch, Sidra Medicine, Doha 26999, Qatar; Computational Sciences Department, The Jackson Laboratory, Farmington, CT 06032, USA; B Lymphocytes, Autoimmunity and Immunotherapies, UMR 1227, Univ Brest, Inserm, Brest 29200, France; Brest University Hospital, Brest 29200, France; Department of Nephrology, AP-HM, Marseille 13003, France; C2VN, INSERM, INRAE, Aix-Marseille Universite, Marseille 13003, France; Translational Medicine Division, Research Branch, Sidra Medicine, Doha 26999, Qatar; Department of Infectious Diseases, St Jude’s Children Research Hospital, TN, Memphis 38105, USA

## Abstract

**In the field of complex autoimmune diseases such as systemic lupus erythematosus (SLE), systems immunology approaches have proven invaluable in translational research settings. Large-scale datasets of transcriptome profiling have been collected and made available to the research community in public repositories, but remain poorly accessible and usable by mainstream researchers. Enabling tools and technologies facilitating investigators’ interaction with large-scale datasets such as user-friendly web applications could promote data reuse and foster knowledge discovery. Microarray blood transcriptomic data from the LUPUCE cohort (publicly available on Gene Expression Omnibus, GSE49454), which comprised 157 samples from 62 adult SLE patients, were analyzed with the third-generation (BloodGen3) module repertoire framework, which comprises modules and module aggregates. These well-characterized samples corresponded to different levels of disease activity, different types of flares (including biopsy-proven lupus nephritis), different auto-antibody profiles and different levels of interferon signatures. A web application was deployed to present the aggregate-level, module-level and gene-level analysis results from LUPUCE dataset. Users can explore the similarities and heterogeneity of SLE samples, navigate through different levels of analysis, test hypotheses and generate custom fingerprint grids and heatmaps, which may be used in reports or manuscripts. This resource is available via this link**: https://immunology-research.shinyapps.io/LUPUCE/. **This web application can be employed as a stand-alone resource to explore changes in blood transcript profiles in SLE, and their relation to clinical and immunological parameters, to generate new research hypotheses**.

## Introduction

There is a need for personalized medicine in systemic lupus erythematosus (SLE), a heterogeneous chronic autoimmune disease (1). The complexity of the interferon (IFN) and other signatures of interest in SLE is still preventing the use of dedicated biomarkers to assess prognosis and/or disease activity and/or to identify patients needing and potentially benefiting of targeted therapeutics (2–7). Tools are needed to enhance immune profiling capabilities in affected patients. Here, we aimed to develop a widely available open access user-friendly application allowing researchers and clinicians to navigate through modular transcriptomic signatures in a well-characterized cohort of adult SLE patients. Indeed, even data deposited in public repositories such as NCBI’s Gene Expression Omnibus (GEO) are not easily accessible and require significant pre-processing before use. A collective effort from our teams and many others aims at permitting re-use of existing data by other investigators, who may employ different analytic approaches or combine these data with additional datasets and perform meta-analyses on large number of samples (8–11).

Here, we utilized blood transcriptome data obtained from the LUPUCE study cohort (NCT00920114), publicly available in GEO (GSE49454). Additionally, we employed comprehensive metadata corresponding to the samples to facilitate the comparison and distinction of blood transcriptomic profiles among adult patients diagnosed with SLE exhibiting diverse clinical and immunological characteristics.

## Materials and methods

The LUPUCE reference transcriptome dataset was generated as follows: transcript abundance was measured via Illumina HumanHT-12 v3.0 Gene Expression BeadChips in 157 samples collected at consecutive available time points from 62 SLE patients, as well as healthy controls. Normalization of the microarray data was performed with the ‘normalize.quantiles’ function from the preprocessCore package. A detailed description of the cohort along with the methodologies used for sample and data processing has been published earlier (4, 5). For the present application, extensive metadata associated with samples were provided, including auto-antibody profiles, disease activity, SLE disease activity index (SLEDAI), types of flares and lupus nephritis classes in patients sampled at the time of kidney biopsy. We also indicated whether the samples had an absent, mild, moderate or strong IFN signature (0, 1, 2 or 3 IFN modules activated according to the generation 2 of modules) as defined in our previous publications (4, 5).

We performed analyses at the module-level and at the module-aggregate-level, using the ‘BloodGen3’ framework of analysis, as previously described (8). The ‘BloodGen3’ repertoire comprises 382 modules, grouped in 28 aggregates. We used the specific corresponding R package called ‘BloodGen3Module’ to perform downstream module-based analyses in the LUPUCE cohort (12).

We developed an R Shiny web application as a user-friendly interface for deploying the LUPUCE app, which can be found at GitHub. This web application consists of the standard Shiny framework components [shinyApp (ui, server)], allowing users to deploy the app directly to their own Shiny accounts. Designed to specialize in the visualization of blood transcriptomic data, the application was built within the R Shiny framework.

To create an intuitive user experience, we first sketched out a wireframe that mapped the application’s user interface. This design includes input fields for data uploads, dropdown menus for selecting various analytical options and areas to display the output results. Leveraging the capabilities of the BloodGen3Module package, we integrated server-side logic in R to support module-level group comparison analyses. The results of these analyses are visualized as annotated fingerprint grid plots. Additionally, the app offers users the option to conduct analyses on individual samples, presenting these results as fingerprint heatmaps.

Upon successful validation, the application was deployed on a Shiny server and accompanied by comprehensive user documentation. This web application serves as an accessible tool for researchers to visualize and interpret changes in blood transcript abundance across various pathological and physiological states in SLE.

Users can access the results of these analyses through the LUPUCE BloodGen3 web application, which is deployed as an R Shiny app and can be accessed at: https://immunology-research.shinyapps.io/LUPUCE/. This application allows users to generate custom plots for use in reports and publications. Additionally, it includes extensive annotations to aid in the interpretation of the data, which can be accessed via different tabs on the left side of the interface. The features of this resource application are presented in more detail below.

## Functional overview

**Figure 1.**
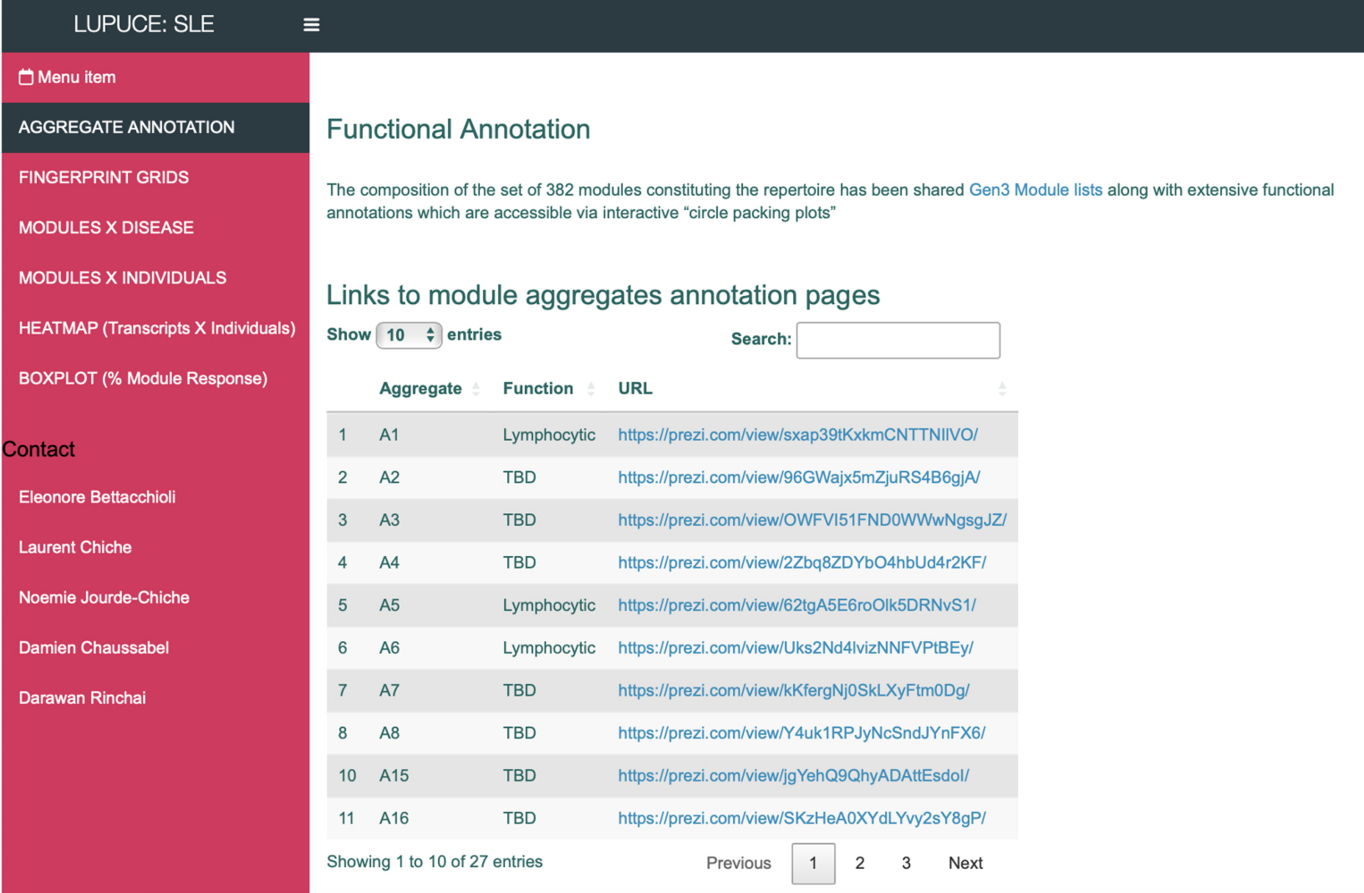
BloodGen3 application user interface.

**Figure 2.**
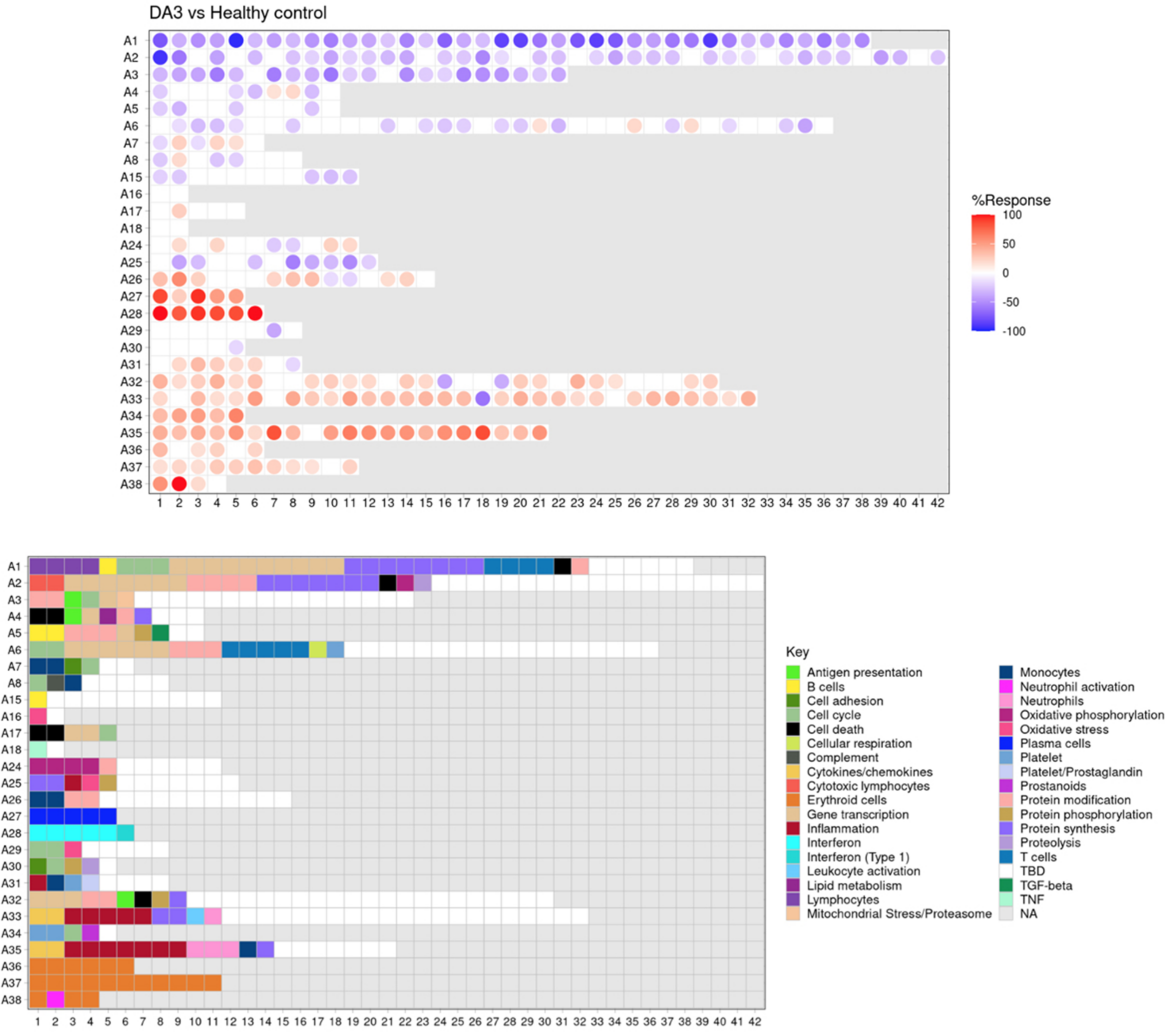
Fingerprint grid plot representation.

**Figure 3.**
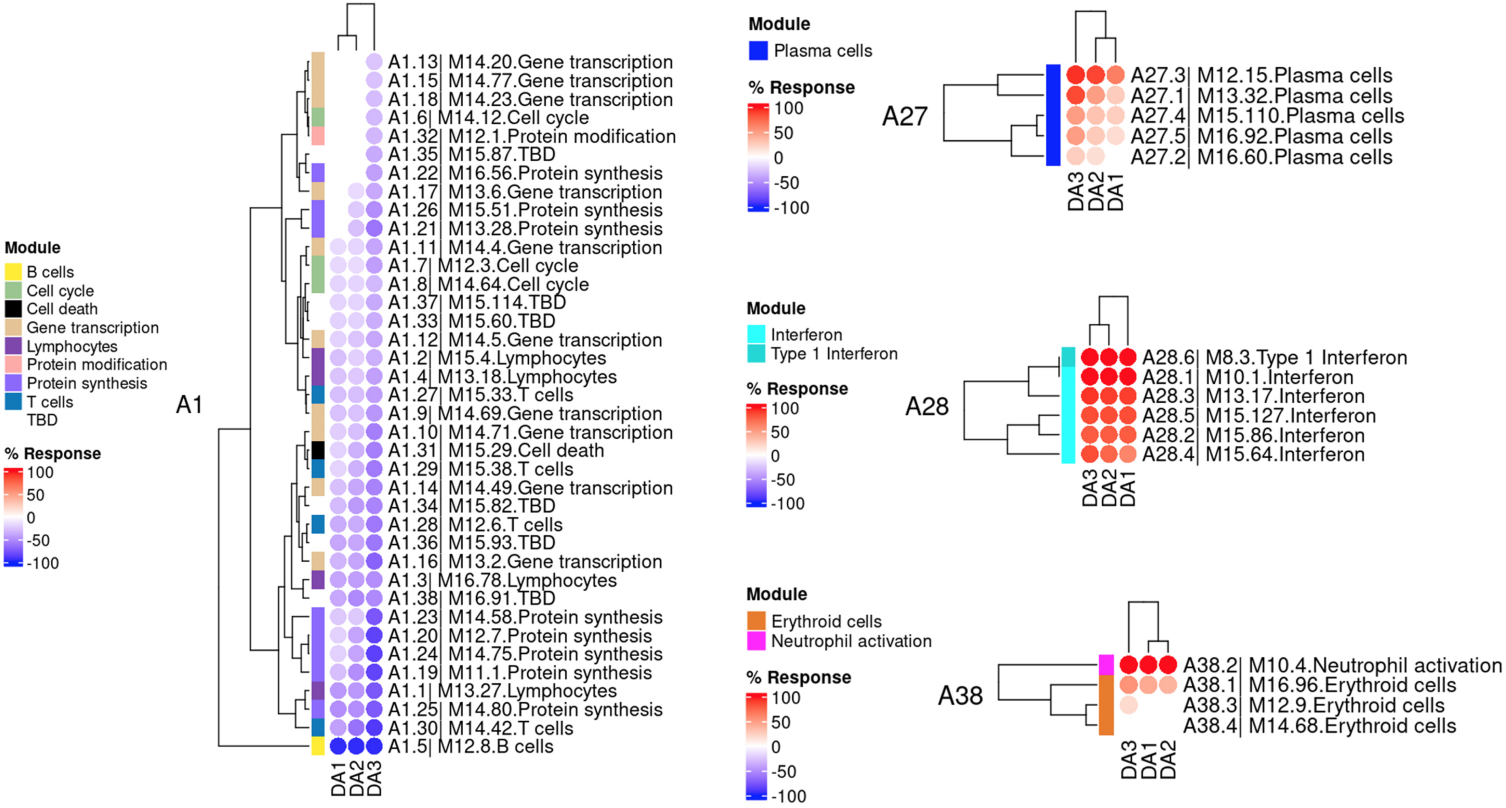
Group-level fingerprint heatmap representation.

**Figure 4.**
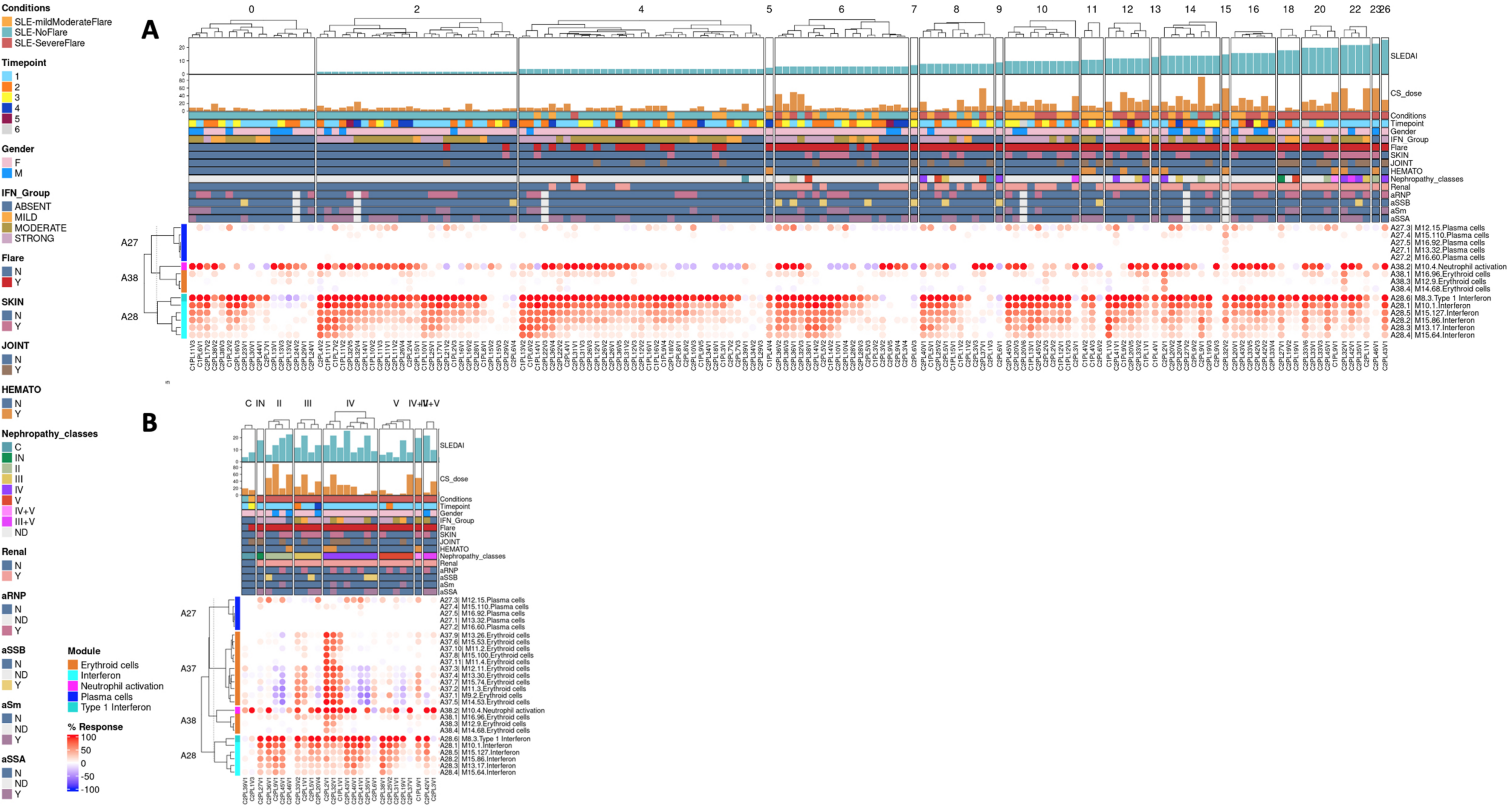
Individual-level fingerprint heatmap representation.

**Figure 5.**
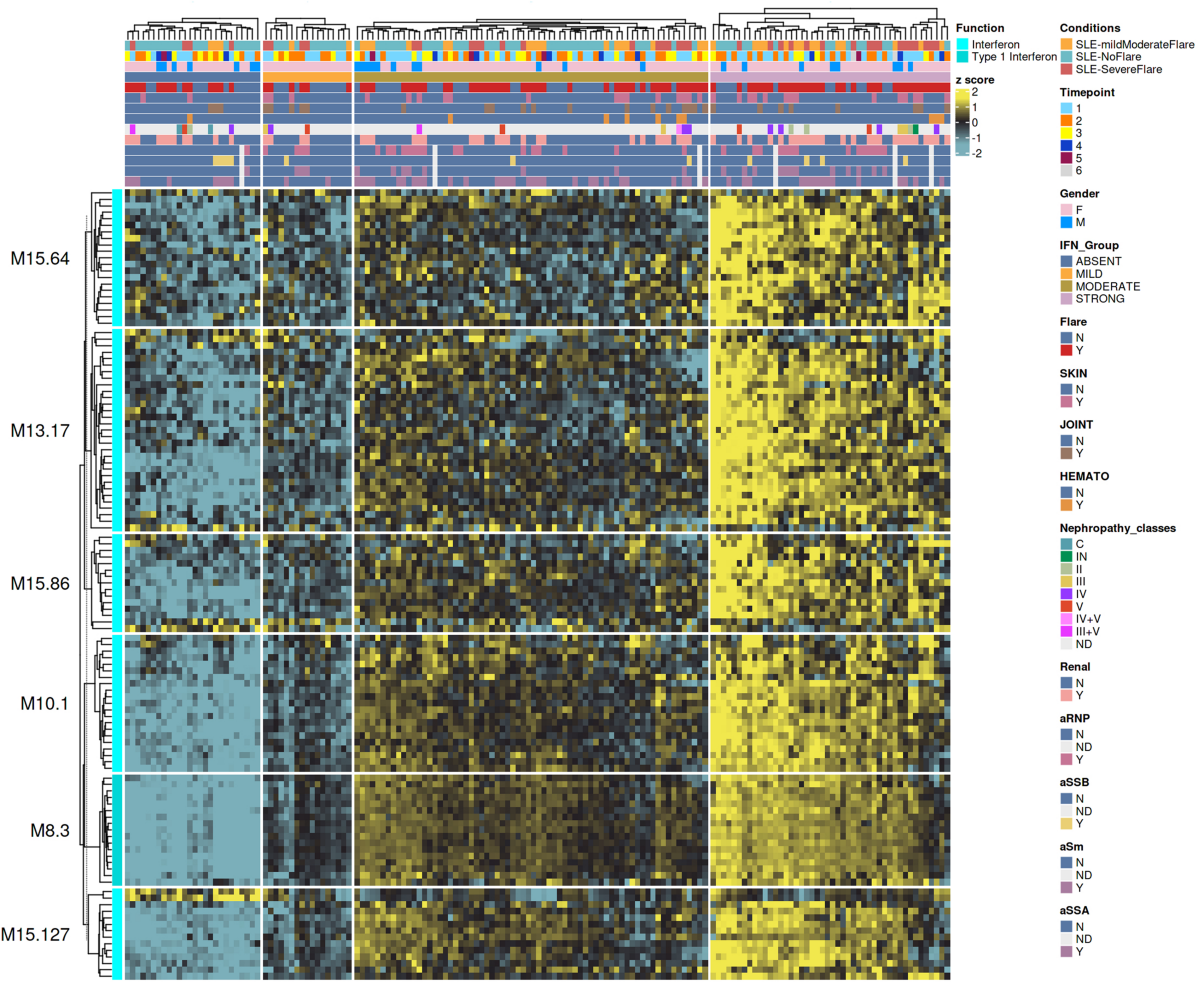
Transcript-level fingerprint heatmap representation according to interferon subgroups.

**Figure 6.**
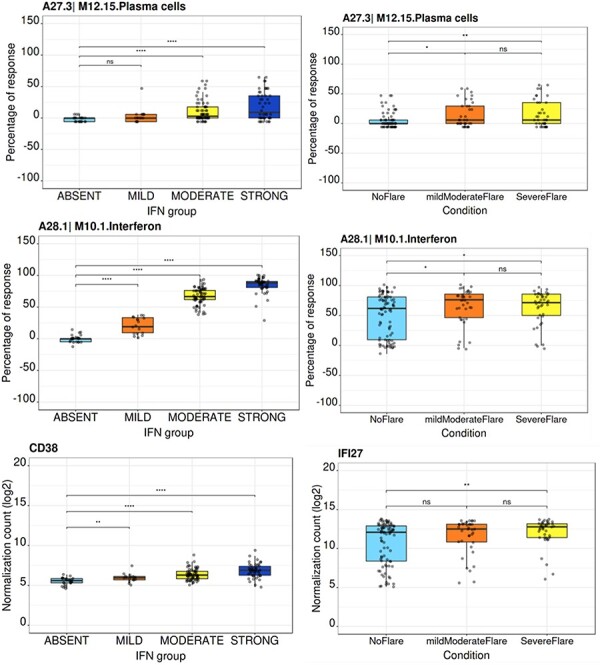
Module activity and gene transcript boxplots representation.

- The ‘**AGGREGATE ANNOTATION**’ tab lists the 28 module aggregates that are used to generate fingerprint grid heatmaps or boxplots (Figure 1). Each module aggregate comprises several modules. Clicking on the links provided, an interactive Prezi presentation can be opened in a new browser window; for instance, in the case of module aggregate A28: https://prezi.com/view/sSTVHAGUMNgkGiNhSbgD/. Clicking on individual modules will permit to zoom in and access background information about the module (individual gene composition), functional profiling information (ontology profiling, pathway and literature enrichment tools, transcription factor binding motif enrichment) and transcriptional profiles for the gene set constituting the module across several reference datasets (isolated leukocyte populations and hematopoietic precursors). These features are illustrated by a screencast video deposited in Figshare (13), also accessible via this link: https://youtu.be/jF1w5DXbr_s.
- The ‘**FINGERPRINT GRIDS**’ tab provides access to fingerprint grid plots which indicate changes in transcript abundance of SLE patients across the LUPUCE dataset based on their disease activity, with the designation DA1 corresponding to no flare, DA2 to mild or moderate flare and DA3 to severe flare, in comparison to healthy controls (Figure 2). The position of the modules on the grid is fixed, with the modules in the same row belonging to the same aggregate. The number of modules per aggregate varies between 2 (aggregate A16) and 42 (aggregate A2). Red spots indicate that a proportion of the transcripts constitutive of the corresponding module have significantly higher abundance levels in SLE patients compared to healthy controls, while blue spots indicate the opposite. The colors are gradated to indicate the percent difference between upregulated transcripts and downregulated transcripts, with values ranging from +100% (all constitutive transcripts are upregulated compared to healthy controls) to −100% (all constitutive transcripts are downregulated compared to healthy controls). An annotated map is provided below that uses a color code to represent the functional annotations associated with each of the modules on the map (no color means that functional associations for these modules have not yet been identified). A short screencast video deposited in Figshare (14) and demonstrating the generation of fingerprint grids based on disease activity can be accessed via this link: https://youtu.be/vgSHNJt-kOk.
- The ‘**MODULES X DISEASE**’ tab provides users access to fingerprint heatmap plots, for each of the aggregates and across the LUPUCE study (Figure 3). The position of the modules is set according to similarities in abundance patterns through hierarchical clustering. In this case, columns on the heatmap correspond to study groups, namely DA1, DA2 and DA3 as mentioned above, and rows correspond to individual modules. The proportion of transcripts for which abundance is significantly changed is displayed using gradated red and blue dots, as previously detailed. Users can access heatmaps for each aggregate by using the drop-down list above the plot (‘Choose aggregate’). Additionally, the zoom in/out function of the web browser can be used to increase the size of the image, thus improving its resolution. The image can then be saved for use in reports or manuscript preparation. A screencast video showcasing these functionalities in action on the application is deposited in Figshare (15) and can be accessed through this link: https://youtu.be/Yudmt7fJaXM.
- The ‘**MODULES X INDIVIDUALS**’ tab provides users with the opportunity to generate custom fingerprint heatmap plots. Rows represent modules for a chosen aggregate, but this time columns represent individual subjects instead of study groups as in the previous tab (Figure 4). Users have the possibility to combine multiple module aggregates by typing in the IDs of the modules of interest (for example, A28 is the ID for module aggregate A28) into the designated box, and can also choose to classify patients according to various clinical and biological features, for example SLEDAI (16), an international scoring system stratifying SLE patients based on disease severity, but also renal involvement, daily dose of corticosteroid taken by patients or auto-antibody serological status. The multiple features of this tab are exemplified in a screencast video that has been deposited in Figshare (17) and can be accessed through this link: https://youtu.be/sRfg0PvjB30.
- The ‘**HEATMAP (TRANSCRIPT X INDIVIDUALS)’** tab provides users access to fingerprint heatmap plots of each transcript contained in modules from a selected module aggregate and according to individual subjects (Figure 5). A drop-down menu called ‘aggregate’ allows the user to choose between each module aggregate (e.g. A28 for the module aggregate A28) in order to display the level of activation of transcripts that compose each constitutive module of the given module aggregate. In this tab, each patient is already clustered according to their IFN subgroup, i.e. ‘absent’, ‘mild’, ‘moderate’ or ‘strong’ IFN signature, based on previous work (4). This feature enables users to gain deeper insight into the characterization of individual patients based on their interferon signature that is linked to many clinical manifestations occurring in SLE patients (2, 4, 18–20). The corresponding screencast video is deposited in Figshare (21) and is available via this link: https://youtu.be/jY9XI9DWvdM.
- The ‘**BOXPLOT (% Module Response)**’ tab provides access to box plots showing the percentage response for individual modules as well as normalized counts of any transcript across study groups of the LUPUCE dataset (Figure 6). In the first section, every module can be selected from a drop-down menu. On the second section, transcripts can be selected from a drop-down menu called ‘Gene symbol’. To facilitate the search for a particular transcript, it is possible to type the first letters of the transcript to get a suggestion from the tool. Results are generated systematically based on (i) IFN groups, which include ‘absent’, ‘mild’, ‘moderate’ and ‘strong’ (4); and (ii) disease activity groups, which include DA1, DA2 and DA3 as presented earlier. A screencast video illustrating the different types of boxplots that can be generated has been deposited in Figshare (22) and is available via the following link: https://youtu.be/NuyiLhDzjzQ.

The BloodGen3 application’s user interface allows users to access various information and visual representations of results through the tabs on the left side. The interface allows for customization of the plots through the use of drop-down menus and sliders, and the resulting plots can be downloaded for use in reports or publications.

The fingerprint grid plot illustrates changes in the abundance of blood transcripts in samples collected from patients with SLE of the LUPUCE study, according to their disease activity. DA1 corresponds to quiescent lupus patients, DA2 to mild lupus flares and DA3 to severe lupus flares. Positions of the different modules on the grid are fixed, with each row grouping modules from the same ‘module aggregate’ labeled A1, A2, A3, etc. Only aggregates with more than one module are shown on the grid (8). Increases in transcript abundance ranging from +15% to +100% are indicated by red spots and decreases from −15% to −100% by blue spots, with the shade of the color indicating the degree of change. This color gradation represents the ‘module activity’, which is the proportion of transcripts that meet a statistical cutoff of *P < 0.05* and FDR <0.1. Here, samples from patients with severe flares (DA3) are compared with samples from healthy controls.The grid below uses a color code to indicate the functional annotations assigned to each module. White areas indicate modules without clear functional associations (TBD) and grey areas indicate unassigned modules (NA).

This heatmap represents changes in transcript abundance for individual modules (rows) of a given aggregate (A1, A27, A28 and A38 in this example) in SLE patients from the LUPUCE study (columns). The columns represent the different categories in terms of disease activity with DA1 being quiescent lupus patients, DA2 being mild lupus flares and DA3 being severe lupus flares. Rows and columns are arranged via hierarchical clustering, based on similarities in abundance profiles. Red spots indicate an increase in transcript abundance compared to the baseline, with proportions ranging from 15% to 100%, as in A27, A28 and A38. Blue spots indicate a decrease in transcript abundance with proportions ranging from −15% to −100%, as in A1. A color code on the vertical annotation track is used to indicate functional associations for the modules shown on the heatmap.

The heatmaps generated in this tab represent changes in transcript abundance for individual modules of multiple aggregates displayed in rows across individual SLE samples (columns). These heatmaps can be customized according to a wide range of categories, including for example the SLEDAI scoring system (16) or the histopathological classification of lupus nephropathies (26), as shown here in panels A and B (see specific legend below). Rows are arranged by hierarchical clustering based on similarities in abundance profiles first across module aggregates and then within module aggregates. Red spots indicate an increase in transcript abundance with proportions ranging from 15% to 100%, and blue spots indicate a decrease in transcript abundance with proportions ranging from −15% to −100%. A color code on the vertical annotation track is used to indicate functional associations for the modules shown on the heatmap as well as the different characteristics of the patients.(A) Individual profiles of module aggregates A27, A28 and A38 are displayed here according to the SLEDAI scoring system (16).(B) Individual profiles of module aggregates A27, A28, A37 and A38 are displayed here specifically among patients with lupus nephritis sampled at the time of kidney biopsy, according to the histological class of lupus nephropathies (26).(C) Chronic lesions only. IN: interstitial nephritis without glomerulonephritis.

This heatmap represents changes in transcript expression (rows) belonging to several modules from one defined module aggregate (here, A28) in relation to each SLE individual (columns). Rows are hierarchically clustered according to the similarity of the expression patterns among the transcripts first across the different modules and then within each module. The columns are already arranged based on the IFN group patient membership, with the groups being ‘absent’, ‘mild’, ‘moderate’ and ‘strong’, according to our previous description in the literature (4). Each cell of the heatmap represents the relative expression level of a specific transcript in an individual SLE patient and its color indicating the level of increase (yellow) or decrease (blue) in comparison to baseline. A color code on the vertical annotation track is also used to indicate the different characteristics of the patients. The heatmap can be used to identify patterns and trends in transcript expression levels across the different IFN subgroups of patients.

The boxplots from the section ‘Module’ represent activity profiles measured as ‘percentage of response’ (y-axis) for individual modules in the LUPUCE dataset. This percentage of response estimates the proportion of constitutive transcripts for which abundance levels are significantly different compared to baseline. Profiles are shown for 2 of the 382 available modules that constitute the BloodGen3 repertoire (8). The boxplots from the section ‘Gene symbol’ represent the normalized count (log2) of a selected transcript (here, CD38 and IFI27, on the y-axis) across all SLE patients. Each boxplot is generated according to two different settings (x-axis): (i) IFN groups, with ‘absent’, ‘mild’, ‘moderate’ and ‘strong’ subgroups (4), and (ii) disease activity groups, with ‘no flare’, ‘mild flare’ and ‘severe flare’ subgroups.An unpaired t-test was employed for each group comparison. Statistical significance levels are indicated as follows: *P < 0.05, **P < 0.01, ***P < 0.001.

## Conclusion

In conclusion, while vast amounts of systems-scale profiling data are available in public repositories, it is not always readily accessible or interpretable. The free open-access web application proposed here is meant to fill this gap and complement our GEO deposition of the primary transcriptomic dataset of our LUPUCE study. Practically, this resource is being employed by our team to support the design of targeted transcript panels and assays for the monitoring of SLE patients. The resource is also being used to generate figures for reports and peer-review publications. Such tools are meant to support the interpretation of large-scale profiling data, but do not require from participants to carry out hands on analyses. Instead, participants, that may not have any bioinformatics skills but are medical experts or immunologists will focus on the interpretation of the data and will rely on the data browsing application to explore analysis results and to generate custom figures. Finally, it may be worth noting that other ‘BloodGen3’ applications have been made available as companion to earlier publications (23–25). The user interface and functionalities follow a similar scheme, and these can be used as a resource for meta-analyses of different cohorts or across different diseases.

## Data Availability

The blood transcriptome datasets which are accessible via our web application are available from the NCBI GEO and SRA repositories, with identifiers GSE49454.
