# Correction to: An interactive web application for exploring systemic lupus erythematosus blood transcriptomic diversity

**DOI:** 10.1093/database/baae059

**Published:** 2024-06-24

This is a correction to: Eléonore Bettacchioli *et al*., An interactive web application for exploring systemic lupus erythematosus blood transcriptomic diversity, *Database*, Volume 2024, 2024, baae045, https://doi.org/10.1093/database/baae045

The article has been updated to acknowledge financial support from the Qatar National Library.

